# Adolescent depression recognition and symptom prediction based on multi-view EEG network: an exploratory study

**DOI:** 10.3389/fpsyt.2026.1908969

**Published:** 2026-07-31

**Authors:** Yanli Zhao, Haitao Chen, Xiaoxiao Ma, Xiaoyue Li, Kai Li, Zihao Wei, Jiaqi Song, Yan Chen, Wen Xin, Guimei Yin, Shuping Tan

**Affiliations:** 1 Psychiatric Research Center, Capital Medical University Affiliated Beijing Huilongguan Hospital, Beijing, China; 2 Laboratory of Brain Science and Intelligent Information Processing, School of Computer Science and Technology, Taiyuan Normal University, Jinzhong, China

**Keywords:** adolescent depression, brain functional network, graph convolutional network, multi-view learning, resting-state electroencephalography

## Abstract

**Background:**

Adolescent depression diagnosis currently relies primarily on subjective self-report questionnaires, with a notable lack of objective neurobiological biomarkers. This study aimed to compare the diagnostic performance of traditional psychological scales with a newly developed Multi-view Adaptive Graph Convolutional Network (MVA-GCN) based on resting-state electroencephalography (EEG), and to exploratorily examine the associations between MVA-GCN-derived brain network features and psychological resilience in adolescents.

**Methods:**

Resting-state EEG data were collected from 44 adolescents with major depressive disorder (MDD) and 30 healthy controls (HCs). The MVA-GCN model integrated three parallel connectivity views—phase-locking value (PLV), Pearson correlation coefficient (PCC), and phase lag index (PLI)—via an adaptive fusion mechanism. We compared the classification performance of questionnaire-based machine learning models against the MVA-GCN, and further examined the correlations between model-highlighted network features and clinical symptom measures, including depression, anxiety, loneliness, rumination, and resilience, with age and sex included as covariates.

**Results:**

Questionnaire-based machine learning models achieved a mean classification accuracy of 86.43%, whereas the MVA-GCN attained 99.84% accuracy in the present dataset. Occipital and fronto-central regions contributed most to the model’s predictions. Increased gamma-band functional connectivity and reduced alpha-band power were identified as potential electrophysiological correlates of group differences. Exploratory correlational analyses at the nominal level (*p* < 0.05, uncorrected) suggested potential group-specific patterns in brain–resilience associations. A *post-hoc* deviation index analysis showed that, within the MDD group, greater deviation from the healthy reference brain–resilience association pattern was significantly correlated with more severe rumination, self-rated depression, clinician-rated depression severity, loneliness, and self-rated anxiety (all FDR *q* < 0.05).

**Conclusion:**

In this exploratory sample, the MVA-GCN demonstrated promising proof-of-concept discriminative capability for adolescent depression compared with traditional self-report scales. However, the brain–resilience association findings were not statistically significant after correction for multiple comparisons and should be interpreted with caution. These results suggest that MVA-GCN-derived network features warrant further investigation in larger, independent cohorts, but do not yet support their use as a clinically validated diagnostic tool.

## 1 Introduction

Depression is a prevalent and debilitating psychiatric disorder characterized by persistent low mood, anhedonia, cognitive impairments, somatic symptoms, and elevated suicide risk, profoundly affecting emotional regulation, social functioning, and quality of life (1, 2). When depression emerges during adolescence, it is of particular clinical concern, as this developmental period is marked by heightened vulnerability to the onset of severe and recurrent mental illness (3).

Adolescence constitutes a critical neurodevelopmental window during which large-scale cortical and subcortical systems undergo extensive reorganization. Longitudinal neuroimaging studies have demonstrated that this process is not confined to a single circuit; rather, it involves heterochronous development across the whole brain, including progressive refinement of association cortices and reorganization of the segregation, integration, and hub architecture of large-scale functional networks (4–6). Concurrently, heightened sensitivity to emotional and social cues, along with ongoing maturational changes in amygdala–prefrontal circuitry, renders adolescents particularly susceptible to emotional dysregulation and stress-related disorders (7, 8).

Notably, emerging evidence suggests that depression-related brain abnormalities during adolescence are likewise distributed across multiple large-scale systems. For instance, unmedicated adolescents with major depressive disorder have been shown to exhibit widespread hypoconnectivity across resting-state networks, coupled with altered connectivity of hub regions such as the dorsal anterior cingulate cortex (9, 10). Meta-analytic findings further implicate aberrant coupling within and between default mode and task-positive/control networks, which are essential for self-referential processing, cognitive control, and emotion regulation (11, 12). Moreover, a recent study reported that salience network volume is enlarged in depression, and this abnormality may even predate overt clinical symptoms, suggesting its potential as a predictive risk marker (13). Collectively, these findings indicate that adolescent depression is best understood not as a disorder of a single brain region, but as a systems-level phenomenon involving widespread network dysregulation. Therefore, objective biomarkers capable of capturing distributed functional network alterations hold promise for early identification and mechanistic investigation.

Electroencephalography (EEG) is a non-invasive, cost-effective neuroimaging technique with millisecond-level temporal resolution, making it well-suited for capturing real-time neurophysiological dynamics. Accumulating evidence supports the utility of EEG in depression research, particularly in revealing individual-level neurophysiological heterogeneity and providing insights into pathophysiological mechanisms (14–16). Importantly, EEG signals can be transformed into graph-based functional connectivity representations, wherein scalp electrodes serve as nodes and connectivity metrics (e.g., phase synchronization or linear correlation) define edges. This approach allows for the explicit modeling of topological relationships among brain regions, thereby offering a systems-level perspective for both clinical assessment and mechanistic inquiry (18).

In recent years, machine learning and deep learning methods have been increasingly applied to EEG-based depression detection. Conventional algorithms, such as support vector machines and random forests, have demonstrated robust performance on modest-sized datasets with well-engineered features. Concurrently, graph-based deep learning approaches, particularly graph convolutional networks (GCNs), have gained traction due to their ability to jointly represent network structure and signal features (17). For example, Moon et al. (19) integrated brain connectivity features with convolutional neural networks for emotion recognition, while Wang et al. (20) proposed a semi-supervised GCN using Pearson correlation-based graphs for depression classification. Other recent studies have explored adaptive graph structures, multi-modal frameworks, and attention mechanisms to further improve classification performance (21–23). Despite these advances, most existing graph-based EEG studies have relied on a single connectivity metric, (e.g., Pearson correlation coefficient, PCC alone) to construct the graph structure, potentially overlooking the complementary information offered by other connectivity measures such as phase synchronization (PLV) or asymmetric coupling (PLI). Moreover, few studies have systematically integrated multi-view connectivity information within an adaptive fusion framework that can dynamically weigh the contribution of each view. To address these gaps, our proposed MVA-GCN framework explicitly incorporates three complementary connectivity metrics—PLV, PCC, and PLI—as parallel views, and introduces an adaptive fusion mechanism to learn view-specific importance weights, thereby enabling a more comprehensive characterization of brain network alterations in adolescent depression.

The specific objectives of this study were threefold: (1) to evaluate whether this multi-view framework improves classification performance compared with conventional EEG-based and questionnaire-based models; (2) to identify the brain regions that contribute most to model prediction using interpretability analysis; and (3) to exploratorily investigate whether MVA-GCN-derived network features show associations with clinically meaningful psychological dimensions, and whether such associations differ between adolescents with MDD and healthy controls. Finally, we assessed whether deviation from a healthy reference brain–behavior association pattern was correlated with symptom burden in depressed adolescents. We hypothesized that the MVA-GCN would achieve superior classification performance compared with single-view and conventional models, and that the model-highlighted network features would show meaningful, though exploratory, associations with psychological resilience and emotional symptoms.

## 2 Methods

### 2.1 Participants

Adolescents with depression were recruited from the outpatient department of Beijing Huilongguan Hospital. Healthy adolescents were recruited in primary and secondary schools and communities. The patient inclusion criteria are as follows: (1) meeting the criteria for a current depressive episode based on the Structured Clinical Interview for DSM-5 (24), and the absence of symptoms of command auditory hallucinations or serious suicidal and self-injurious ideas and behaviors; (2) age between 10 and 18 years; (3) no dyslexia in Chinese; (4) no electroconvulsive therapy or transcranial magnetic stimulation was used in the past six months. The patient exclusion criteria are as follows: (1) patients with nervous system disease, personality disorder, or alcohol and drug abuse in the past six months; (2) patients with schizophrenia spectrum disorder who were diagnosed or suspected; and (3) patients who were impulsive and excited and unable to cooperate with the study. The inclusion criteria for healthy controls were: (1) screened with SCID-I and SCID-II (25) to guarantee that they did not have schizophrenia, schizophrenic affective disorder, depression, anxiety disorder, bipolar affective disorder, or other mental disorders; (2) no family history of mental illness; (3) age and education level requirements consistent with patients; (4) volunteered to participate in the study and signed an informed consent form. All procedures in this study were in accordance with the 1964 Helsinki declaration. The experimental protocol was approved by the Ethics Committee of Beijing Huilongguan Hospital (Ethics Protocol Approval Number: 2019-39-science-Revision 1). All eligible adolescents volunteered to take part in this research, and both participants’ verbal assent and written parental informed consent were obtained prior to data collection.

### 2.2 Clinical assessment

The 24-item Hamilton Depression Rating Scale (HAMD; 26, 27) and 14-item Hamilton Anxiety Rating Scale (HAMA; 28) were administered independently by four trained psychiatrists to assess clinician-rated depressive and anxiety severity, with an inter-rater Cronbach’s *α* of 0.83. Five self-report questionnaires were completed by participants: the 18-item Depression Self-Rating Scale for Children (DSRSC; 29, 3-point Likert: 0–2, higher scores = worse depressive symptoms), the 13-item Chinese Spence Children’s Anxiety Scale (SCAS; 30, 31, 5-point Likert: 1–5, higher scores = greater anxiety), the 20-item Revised UCLA Loneliness Scale (R-UCLA-LS; 32, 4-point Likert: 1–4, higher scores = stronger loneliness), the 22-item Ruminative Response Scale (RRS; 33, 4-point Likert: 1–5, higher scores = intensified rumination), and the 27-item Resilience Scale for Chinese Adolescents (RSCA; 34, 5-point Likert: 1–5, higher scores = superior psychological resilience).

### 2.3 Experimental procedure

The experiment utilized an alternating resting-state paradigm to collect EEG signals from all participants. First, participants were instructed to maintain a seated position in an awake state, with their eyes focused on a fixation point for a 5-minute eyes-open (EO, open-eye resting) resting-state session. This was followed by a 5-minute eyes-closed (EC, closed-eye resting) resting-state session, during which participants relaxed with their eyes closed. To ensure data validity, participants were required to remain still, quiet, and relaxed throughout the experiment, minimizing blinking as much as possible. Real-time electromyographic monitoring was employed to exclude interference from body movements.

### 2.4 EEG data recording and acquisition

**Figure 1:**
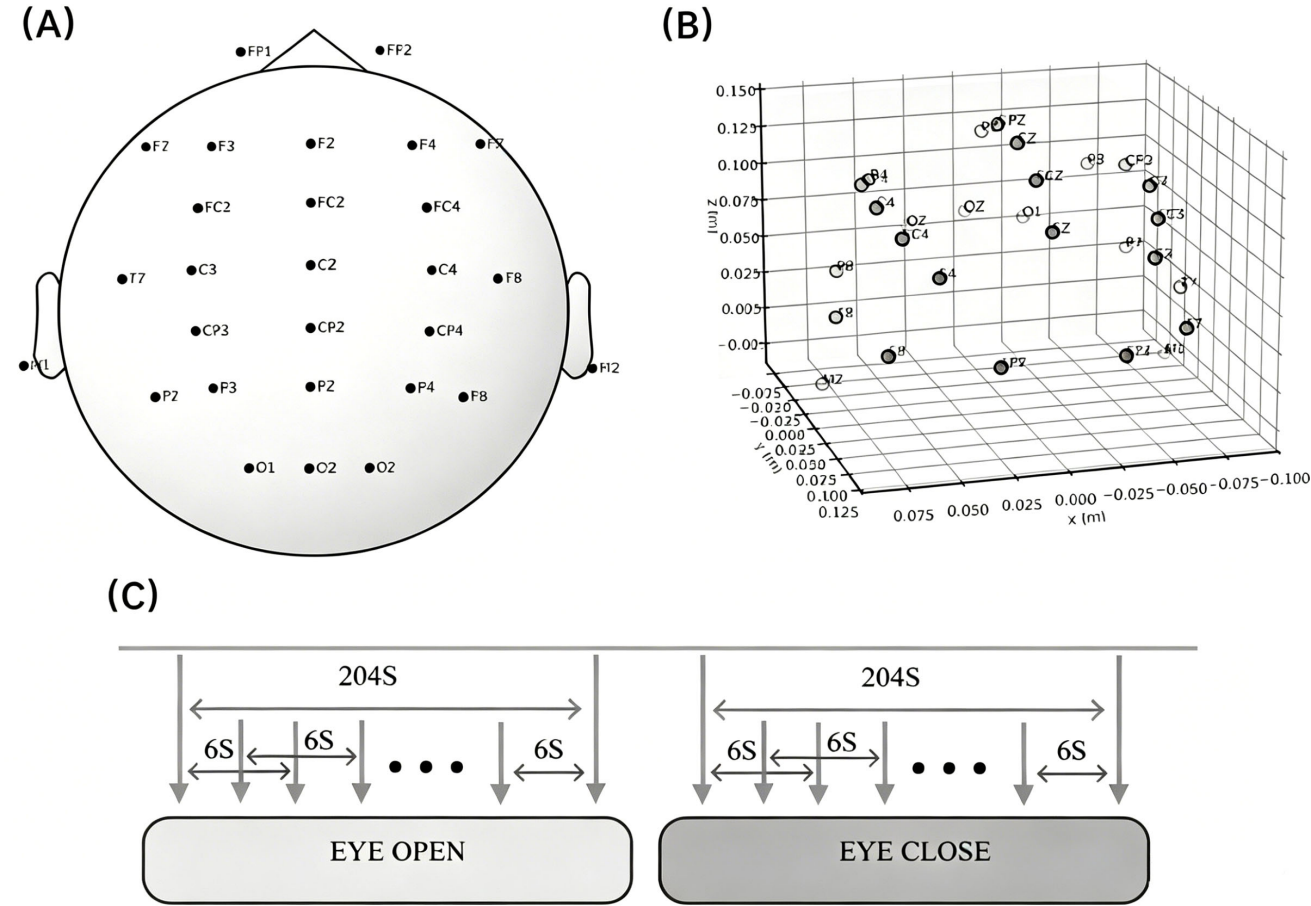
Distribution of scalp electrode locations and sliding-window segmentation pipeline for resting-state EEG preprocessing **(A–C)**.

Electroencephalography (EEG) signals were acquired using a 32-channel electrode cap and the SynAmps RT amplifier system manufactured by Compumedics Neuroscan, following standard international clinical neurophysiology recording protocols (35). Electrode impedance was monitored in real time and maintained below 5 kΩ to ensure signal quality, with the AFz electrode serving as the system ground. Reference electrodes (M1/M2) were physically connected to the left and right mastoids. The sampling frequency was set to 1024 Hz. To minimize ocular artifact contamination, vertical electrooculography (VEOG) electrodes were placed 1 cm above and below the left orbital rim, respectively, while horizontal electrooculography (HEOG) electrodes were symmetrically attached 1 cm lateral to the outer canthus of the right eye. As illustrated in **Figures 1A,B**, after excluding unused electrodes, a total of 28 electrode channels (FP1, FP2, F7, F3, FZ, F4, F8, FC3, FCZ, FC4, T7, C3, CZ, C4, T8, CP3, CPZ, CP4, M1, M2, P7, P3, PZ, P4, P8, O1, OZ, O2) were retained to construct the functional network nodes.

### 2.5 EEG data preprocessing

EEG data preprocessing was performed using the open-source Python toolbox MNE-Python (version 1.4.0; 36), which is specifically designed for EEG/MEG signal processing, analysis, and visualization (37). After data import, the signals were first resampled to 500 Hz. Horizontal electrooculography (HEOG), vertical electrooculography (VEOG), Trigger channels, and other non-target channels not used in subsequent analyses were then removed, ultimately retaining 28 EEG channels as functional network nodes. Electrode positions were matched according to the international 10–20 standard system.

Bad channels were identified through visual inspection. Specifically, channels were marked as bad if they exhibited persistently flat signals or abnormal baseline drift. Marked bad channels were then repaired using spherical spline interpolation. After data repair, the data were re-referenced to the common average reference, followed by band-pass filtering at 0.5–50 Hz (zero-phase FIR filter, 24 dB/oct roll-off) and notch filtering at 50 Hz to remove low-frequency drift and power-line interference. Following filtering, data segments containing large-amplitude artifacts (e.g., muscle artifacts, eye movements, eye blinks) were independently identified and rejected via visual inspection on the continuous data by two experienced researchers, with disagreements resolved through discussion. The proportion of rejected segments accounted for less than 3% of the total continuous data across all participants, with the majority of participants showing rejection rates below 2%.

For fine-grained artifact correction, an independent component analysis (ICA)-based artifact correction approach was adopted. The extended Infomax algorithm (38) was employed for ICA decomposition. Components potentially containing artifacts were manually inspected and removed primarily based on their spatial topographies and time-series characteristics. Ocular-related components typically exhibited prominent frontal distributions and blink- or eye-movement-like time courses, whereas muscle-related components tended to show more focal distributions, higher-frequency activity, and irregular temporal waveforms. Based on retrospective inspection of the processing logs, approximately 4–6 ICA components were removed per participant, consistent with typical EEG preprocessing practices for this channel configuration. After ICA correction, automatic amplitude detection (threshold: ± 80 μV) was applied to identify any residual large-amplitude artifacts, followed by a second manual visual inspection to ensure data purity. Ultimately, the data retention rate exceeded 95% for each participant, and thus all participants were included in the subsequent analyses.

Following preprocessing, each participant’s EEG data was segmented according to the markers for the eyes-open (EO) and eyes-closed (EC) conditions. To avoid the influence of initial unstable states, EEG data from 48 to 253 seconds after recording onset (approximately 204 seconds of stable data) were extracted separately for the EO and EC conditions. The extracted data were then segmented using 6-second sliding windows with 50% overlap, corresponding to a step size of 3 seconds. All windows passed the above amplitude detection criteria. Each EO or EC condition yielded 67 data segments; thus, each participant contributed 134 EEG epochs in total. These epochs were subsequently used for multi-frequency band decomposition, functional connectivity matrix construction, and multi-view aggregation graph convolutional network (MVA-GCN) model analysis. Frequency band decomposition was performed using the short-time Fourier transform (STFT) method, and the data were divided into five sub-bands according to classical frequency bands: Delta (1–3 Hz), Theta (4–7 Hz), Alpha (8–12 Hz), Beta (13–30 Hz), and Gamma (31–49 Hz).

### 2.6 Experimental environment and parameter settings

All experiments in this study were implemented using the PyTorch deep learning framework, with model training conducted on an NVIDIA RTX 4060 GPU.

The model consists of two graph convolutional layers and a fully connected classifier. The first graph convolutional layer has 16 output channels, the second graph convolutional layer has 4 output channels, and a fully connected layer with 28 dimensions follows. The model training process employs the Adam optimizer with an initial learning rate of 0.001, which decays by a factor of 10 as the number of iterations increases. The dropout rate for each layer is set to 0.2, and the cross-entropy loss function is used. Training is performed for 100 epochs.

### 2.7 Statistical analyses

For baseline between-group comparisons, continuous variables were analyzed using Welch’s t-test, and categorical variables were analyzed using Fisher’s exact test. Unless otherwise specified, all regression analyses in this study were conducted using complete-case data.

To examine the potential clinical relevance of the MVA-GCN network, the study was conducted at four analytical levels.

First, in the disease classification task, the performance of EEG-based prediction models derived from brief resting-state EEG recordings was compared with that of questionnaire-based prediction models, and the performance of MVA-GCN was further compared with that of other EEG-based models. Accuracy (Acc), precision (Pre), recall (Rec), F1, and the area under the receiver operating characteristic curve (AUC) were used as the primary evaluation metrics.

Second, the Grad-CAM algorithm (39) was used to identify nodes that contributed substantially to depression classification. To improve interpretability, these model-relevant nodes were grouped into a posterior region and an anterior-central region according to spatial proximity. Degree and betweenness were selected as theoretically central and widely used topological metrics with clear interpretive relevance (40, 41). Specifically, degree reflects the extent of a node’s connections with other nodes, whereas betweenness reflects the intermediary or hub-like role of a node in information transmission pathways.

Third, to examine the associations between MVA-GCN network features and clinical scales, and to test whether these associations differed between the depressed and healthy control groups, linear regression models were constructed with clinical scales as dependent variables and network features as predictors, with additional group-by-network feature interaction terms included in the models. Because the final interpretability analysis focused on 2 regions and 2 features, false discovery rate (FDR) correction was applied within each scale across the four interaction tests (2 regions × 2 features), and adjusted q values were reported (42).

Fourth, to further evaluate higher-order correspondences between the key brain network features identified by the MVA-GCN model and clinical manifestations, a healthy reference model was established in the healthy control group, and a deviation index was then calculated in the depressed group as the predicted value minus the observed value; higher values indicated greater deviation from the healthy reference association pattern. FDR correction was applied across patient scale outcomes, and adjusted q values were reported (42).

All of the above models included age and sex as covariates. All statistical analyses were two-tailed, with the significance level (α) set at 0.05. All statistical calculations adopted complete-case analysis without data imputation; missing data occurred for HAMD (1 case), HAMA (3 cases), and SCAS (1 case), and these incomplete samples were excluded only from analyses involving the corresponding scales.

## 3 Construction and clinical validation of the MVA-GCN

EEG signals originate from the synchronous generation of postsynaptic potentials in neuronal assemblies, characterized by high-precision temporal properties. They directly reflect neural activity at the population level in the human brain, enabling non-invasive and systematic analysis of brain electrophysiological dynamics, thereby providing crucial methodological support for investigating mechanisms underlying cognitive dysfunction and neuropsychiatric disorders. (43).

Functional connectivity in the brain is defined as the statistical correlation between signals from different brain regions. Brookes et al. further specified functional connectivity as the temporal correlation of neural oscillation amplitude envelopes within specific frequency bands. (44) Previous studies have utilized scalp electrode channels as nodes and employed phase synchronization metrics such as PLV (45), PLI (46) and PCC (47) to calculate connectivity edges between nodes, thereby constructing functional connectivity matrices.

This study proposes a Multi-View Adaptive Graph Convolutional Network model, referred to as MVA-GCN. The term “multi-view” refers to the integration of three distinct functional connectivity metrics—the phase locking value (PLV), Pearson correlation coefficient (PCC), and phase lag index (PLI)—as separate views of the brain network. Each view captures a different aspect of inter-regional interactions: PLV reflects phase synchronization, PCC captures linear correlations, and PLI describes asymmetric coupling. These views are processed independently through graph convolution and then adaptively fused. Importantly, this framework does not involve 3D convolution; rather, each connectivity view is processed independently through graph convolution before adaptive fusion. The core innovation of this model lies in its ability to integrate adjacency matrices constructed from these three functional connectivity metrics into a unified multi-view graph convolutional framework through multi-view graph convolution and an adaptive fusion mechanism. By performing multi-view graph convolution operations across the three parallel views, the model simultaneously learns the topological structure of brain networks from multiple perspectives and adaptively fuses these views, enabling a more comprehensive and precise extraction of brain network information.

### 3.1 Model framework

**Figure 2:**
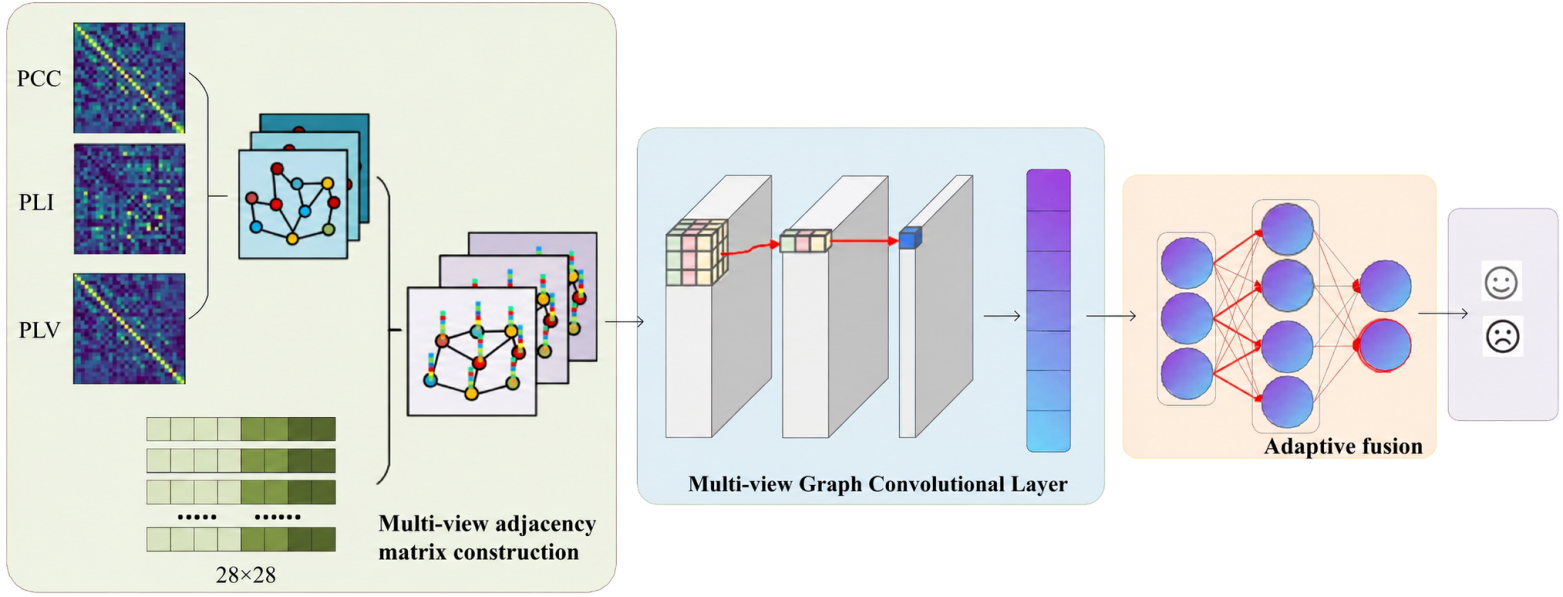
Schematic architecture of the proposed Multi-view Adaptive Graph Convolutional Network (MVA-GCN). The model integrates three parallel connectivity views (PLV, PCC, and PLI), each processed independently through graph convolution, followed by adaptive fusion and classification.

As shown in **Figure 2**, the overall framework of MVA-GCN is divided into the following four modules:

1. Multi-view adjacency matrix construction: The three adjacency matrices A_PCC, A_PLV, and A_PLI (each of shape 28 × 28) are stacked to form a multi-view adjacency tensor of shape 3 × 28 × 28, which serves as the input for subsequent multi-view graph convolution operations.
2. Multi-view Graph Convolutional Layer: The multi-view adjacency matrices are stacked into a multi-view adjacency tensor to enable efficient multi-view graph convolution operations. Each view undergoes graph convolution independently to prevent information confusion.

Each view undergoes graph convolution independently to prevent information confusion. The input consists of the multi-view adjacency matrices $A_{PCC},\;A_{PLV}\text{ and }A_{PLI}$​, along with the node feature matrix $X\in {\mathbb{R}}^{28\times 28}$. The node features include 10 time-domain features, 1 frequency-domain feature, and 4 brain network features separately extracted from each of the three functional connectivity matrices. A multi-view graph convolution kernel is designed to perform graph convolution operations independently for each view (Equation 1)

(1) $$H_{v}^{\left(l+1\right)}=\sigma ({\tilde{D}}_{v}^{-\frac{1}{2}}{\tilde{A}}_{v}{\tilde{D}}_{v}^{-\frac{1}{2}}H_{v}^{\left(l\right)}W_{v}^{\left(l\right)})$$

Among them, ${\tilde{A}}_{v}=A_{v}+I$ is the adjacency matrix of the *v* view with added self-connections, $D_{v}$ ​is the degree matrix, $W_{v}^{\left(l\right)}$​ represents the convolution kernel weights of the *v* view, and $H_{v}^{\left(l\right)}\;$​denotes the node features of the *v* view.

Subsequently, the output features from each view are concatenated, the output features from each view are concatenated (Equation 2) resulting in the multi-view fused feature matrix $H^{\left(l+1\right)}$ with a shape of $28\times 3\hat{D}$, where $\hat{D}$ represents the output feature dimension of each view.

(2) $$H^{\left(l+1\right)}=\text{Concat}(H_{PCC}^{\left(l+1\right)},\;H_{PLV}^{\left(l+1\right)},\;H_{PLI}^{\left(l+1\right)})$$

3. Adaptive fusion mechanism:​ The adaptive fusion mechanism dynamically adjusts the weights of each view, enhancing the model’s sensitivity to important views (Equation 3). The input is the multi-view fused feature matrix $H^{\left(l+1\right)}$, and the weight for each view is computed.

(3) $$\alpha_{v}=\text{Softmax}(\text{MLP}(H_{v}^{\left(l+1\right)}))$$

Among them, MLP stands for Multi-Layer Perceptron, and $\alpha_{v}$​ represents the importance weight of the v view. Subsequently, a weighted summation is performed on the multi-view features (Equation 4), resulting in the fused output feature matrix $H_{fused}$​.

(4) $$H_{fused}=\underset{v\in \{PCC,PLV,PLI\}}{\sum }\alpha_{v}\cdot H_{v}^{\left(l+1\right)}$$

4. Classification module:​ The fused feature matrix $H_{fused}$ ​is input into a fully connected layer, followed by a Softmax function, to produce the classification result y (Equation 5).

(5) $$y=\text{Softmax}(\text{MLP}(H_{fused}))$$

### 3.2 Performance evaluation for depression recognition

To comprehensively evaluate the performance of the depression recognition model, this study employs multiple evaluation metrics, including accuracy (Acc, Equation 6), precision (Pre, Equation 7), recall (Rec, Equation 8), F1-Score (Equation 9), and the area under the curve (AUC). The formulas for each metric are as follows:

(6) $$Acc=\frac{TN+TP}{N}$$

(7) $$Pre=\frac{TP}{TP+FP}$$

(8) $$Rec=\frac{TP}{TP+FN}$$

(9) $$F1=\frac{2\times Pre\times Rec}{Pre+Rec}$$

Where TP represents the number of true positive segments, TN the number of true negative segments, FP the number of false positive segments, FN the number of false negative segments, and N the total number of segments.

AUC denotes the area under the curve; a higher AUC value indicates better classification performance of the model. Through these metrics, the model’s performance in the depression recognition task can be comprehensively evaluated.

A subject-level ten-fold cross-validation strategy was used to evaluate model performance. Specifically, during the training–test split, the data were partitioned at the participant level, ensuring that all EEG epochs from the same participant were assigned either to the training set or to the test set, but not to both. This strategy was adopted to avoid potential data leakage caused by random epoch-level splitting and to provide a more appropriate evaluation of the model’s generalization ability on unseen participants. A total of 44 depressed adolescents and 30 healthy controls were included in this study, resulting in 74 independent participants. For each participant, 67 EEG epochs were generated under the EO condition and another 67 EEG epochs were generated under the EC condition, yielding 134 EEG epochs per participant. Therefore, the final number of epoch-level EEG samples used for model analysis was 74 × 134 = 9,916, including 44 × 134 = 5,896 epochs from the depressed group and 30 × 134 = 4,020 epochs from the healthy control group. Sliding-window segmentation increased the number of model input instances while preserving participant-level EEG information and enabled the model to learn brain network features from different temporal segments.

## 4 Results

### 4.1 Descriptive statistics

A total of 44 adolescents with depression and 30 healthy controls (HCs) were enrolled in this study. No significant difference in sex distribution was observed between the two groups; however, the MDD group was slightly older than the HCs (**Table 1**). Consequently, age and sex were included as covariates in subsequent regression analyses. As anticipated, compared to the HC group, adolescents with MDD exhibited significantly higher scores on the DSRSC, SCAS, R-UCLA-LS, and Rumination Scales, while demonstrating significantly lower scores on the Resilience Scale, indicating a generalized decline in psychological adaptation among depressed adolescents (**Table 1**).

**Table 1:** Demographic information and clinical scale scores of depressed adolescents and healthy controls.

Variable	MDD (*n* = 44)	HC (*n* = 30)	Statistics	*p*-value
Sex, (male/female)	16/28	10/20	/	0.810
Age, year	13.86 ± 2.24	12.40 ± 2.42	2.64	0.011
HAMD	19.00 ± 6.76 (Missing = 1)	/	/	/
HAMA	13.88 ± 5.12 (Missing = 3)	/	/	/
DSRSC	18.82 ± 7.78	7.33 ± 4.92	7.78	<0.001
SCAS	39.33 ± 12.14 (Missing = 1)	22.03 ± 7.91 (Missing = 1)	7.22	<0.001
R-UCLA-LS	56.26 ± 12.13 (Missing = 1)	32.86 ± 10.71 (Missing = 1)	8.49	<0.001
RSCA	26.43 ± 8.98	39.97 ± 8.59	-6.53	<0.001
RRS	58.21 ± 15.18 (Missing = 2)	34.20 ± 7.39	8.73	<0.001
Medication status, n (%)				
Antidepressant (AD) monotherapy	5(11.4%)	0(0%)		
AD + antipsychotics (AP)	4(9.1%)	0(0%)		
AD + Anxiolytics (ANX)	5(11.4%)	0(0%)		
AD + AP + ANX	7(15.9%)	0(0%)		
AD+AP+Mood stabilizers (MS)	1 (2.3%)	0(0%)		
AD + AP + ANX + MS	5(11.4%)	0(0%)		
No psychotropic medication	8(18.2%)	30(100%)		<0.001
Missing data	9(20.5%)	0(0%)		

Values are presented as mean ± standard deviation or n (%). Missing sample quantities are listed in parentheses. Statistical comparisons were conducted via Welch’s t-test for continuous variables and Fisher’s exact test for sex distribution and medication status. Medication classification was based on documented prescriptions at the time of EEG acquisition. Percentages for medication status are calculated based on the 35 patients with complete medication records (rather than the total MDD sample of 44), to accurately reflect the distribution among those with available data.

### 4.2 Performance of the MVA-GCN brain network in adolescent depression recognition

To evaluate the discriminative capability of clinical self-report scales, this study employed various classical machine learning algorithms. The results suggested that the optimal classification performance based on self-report scales achieved an Accuracy (Acc) of 86.43%, Precision (Pre) of 90.17%, Recall (Rec) of 89.00%, F1-score (F1) of 88.70%, and an Area Under the Curve (AUC) of 89.67% (**Table 2**).

**Table 2:** Classification performance of depression prediction using different machine learning models based on self-reported scale data.

Models	Acc	Pre	Rec	F1	AUC
Logistic Regression	81.07 ± 14.78	82.81 ± 12.57	86.00 ± 16.96	83.94 ± 13.77	87.83 ± 11.68
LDA	85.00 ± 13.55	90.17 ± 13.20	86.00 ± 16.96	86.74 ± 12.39	88.83 ± 12.05
QDA	84.82 ± 15.46	87.17 ± 14.45	88.00 ± 17.35	86.88 ± 14.24	89.67 ± 13.67
kNN	86.43 ± 14.36	90.00 ± 14.14	88.50 ± 12.26	88.70 ± 11.38	85.50 ± 13.33
Decision Tree	83.75 ± 15.42	89.17 ± 14.72	84.00 ± 18.83	85.50 ± 14.38	86.00 ± 13.52
SVM	85.00 ± 15.13	88.67 ± 15.73	88.50 ± 12.26	87.81 ± 11.71	89.17 ± 15.02
MLP	85.18 ± 15.93	87.50 ± 14.39	89.00 ± 15.06	87.68 ± 13.15	85.83 ± 15.74

Acc, accuracy; Pre, precision; Rec, recall; F1, F1-score; AUC, area under the curve. All metrics are shown as mean ± standard deviation.

In contrast, the disease prediction model based on 408 s of resting-state EEG showed higher average classification performance across the above metrics. Specifically, the MVA-GCN model achieved average values approaching 99.84% across all measures (Acc: 99.84%, Pre: 99.85%, Rec: 99.84%, F1: 99.84%, AUC: 99.84%). It should be noted that this result reflects the performance of MVA-GCN in the current dataset and under the current ten-fold cross-validation framework. Therefore, it should be interpreted as a preliminary exploratory finding within the present sample, rather than as evidence of fully validated clinical generalizability. Furthermore, the comparisons across different classifiers in **Tables 2**, **3** are descriptive in nature; formal statistical tests between models were not performed, and the observed performance differences should therefore be interpreted with caution.

**Table 3:** Classification performance comparison between MVA-GCN and traditional EEG-based predictive models.

Models	Acc	Pre	Rec	F1	AUC
SVM	85.2 ± 0.7	84.3 ± 0.7	85.1 ± 0.7	84.7 ± 0.7	90.1 ± 0.7
RF	87.6 ± 0.5	86.9 ± 0.5	87.5 ± 0.5	87.2 ± 0.5	92.3 ± 0.5
MLP	89.1 ± 0.4	88.5 ± 0.4	89.0 ± 0.4	88.7 ± 0.4	93.4 ± 0.4
CNN	91.2 ± 0.3	90.8 ± 0.3	91.1 ± 0.3	90.9 ± 0.3	95.1 ± 0.3
GCN	99.0 ± 0.2	98.8 ± 0.2	98.9 ± 0.2	98.8 ± 0.2	99.8 ± 0.2
MVA-GCN	99.8 ± 0.3	99.9 ± 0.3	99.8 ± 0.3	99.8 ± 0.3	99.8 ± 0.3

Acc, accuracy; Pre, precision; Rec, recall; F1, F1-score; AUC, area under the curve. All performance values are reported as percentage ± standard deviation.

### 4.3 Clinical relevance of MVA-GCN brain features in depression

**Figure 3:**
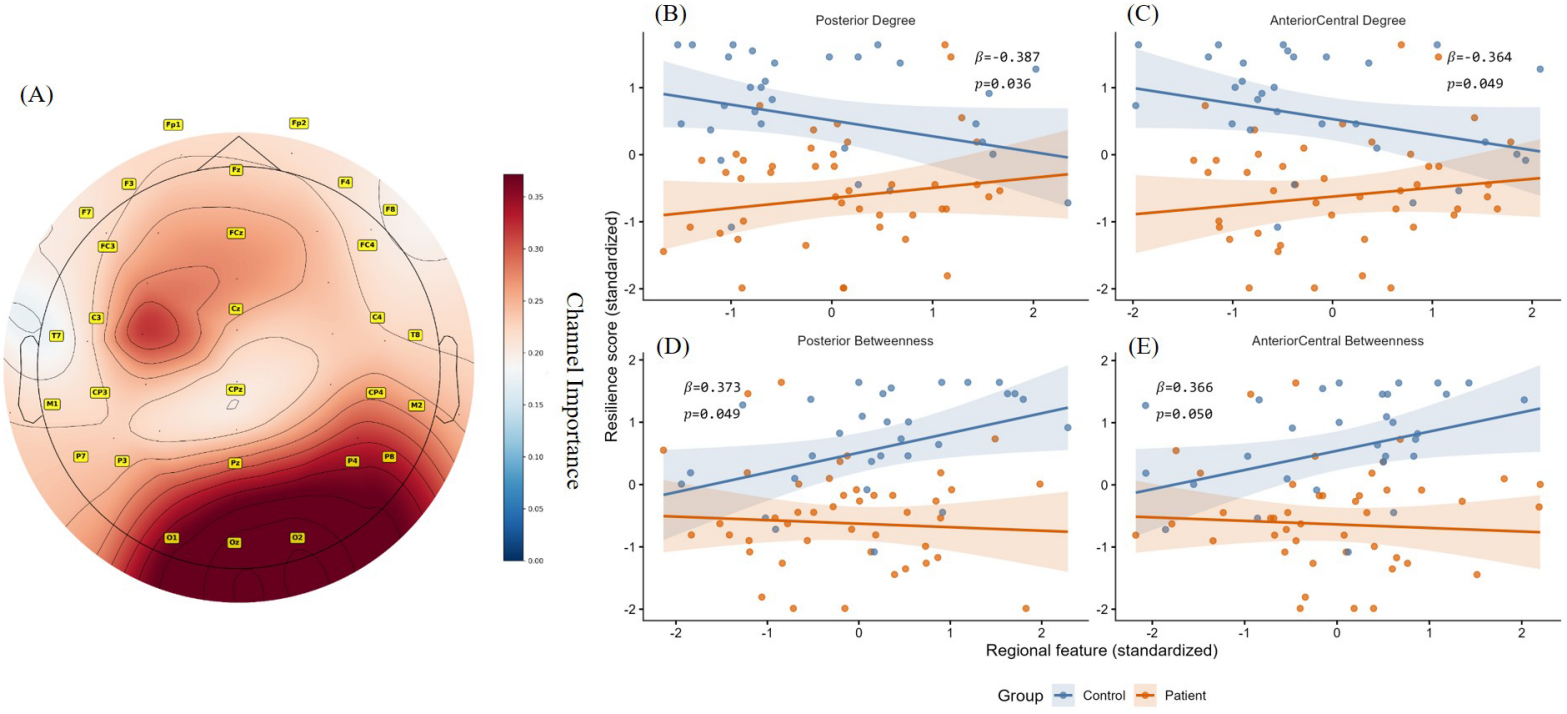
Important brain regions identified by Grad-CAM and group-dependent correlations between nodal network metrics and psychological resilience **(A–E)**.

To elucidate the neurobiological underpinnings of the model’s predictions, we employed the Grad-CAM algorithm (39) to identify the brain region nodes contributing most significantly to the classification of depression. The interpretability analysis revealed that the most influential nodes were predominantly distributed across the occipital and frontal regions, specifically including O2, Oz, O1, P8, C3, FC3, P4, FCz, Fz, and F3 (**Figure 3A**). Based on their physical spatial distribution, these nodes were delineated into two distinct clusters: the anterior central region (C3, FC3, FCz, Fz, F3) and the posterior region (O2, Oz, O1, P8, P4).

To further investigate the clinical implications of the MVA-GCN brain network, we constructed linear regression models with self-report scale scores as dependent variables and Group × ROI feature interactions as predictors, controlling for age and sex as covariates. Following FDR correction, none of the interaction effects reached the conventional threshold for statistical significance (all *q* ≥ 0.050). Specifically, the four resilience-related interaction tests yielded FDR-adjusted q values of 0.050 for all four interactions: Degree of the posterior region (*β* = -0.387, *p* = 0.036, FDR *q* = 0.050), Degree of the anterior central region (*β* = -0.364, *p* = 0.049, FDR *q* = 0.050), Betweenness of the posterior region (*β* = 0.373, *p* = 0.049, FDR *q* = 0.050), and Betweenness of the anterior central region (*β* = 0.366, *p* = 0.050, FDR *q* = 0.050). These results did not survive FDR correction and should therefore not be interpreted as statistically significant; they are reported for transparency and hypothesis-generation purposes only.

At the nominal level (*p* < 0.05, uncorrected), *post-hoc* analyses revealed group-dependent correlational patterns between nodal topological metrics and resilience: nodal degree of anterior-central and posterior brain regions positively correlated with resilience among healthy adolescents, while such correlational patterns were not observed in depressed participants, suggesting potential group-specific decoupling effects (**Figures 3B, C**). Conversely, betweenness centrality in both regions was negatively correlated with resilience in controls but not in patients (**Figures 3D, E**). Importantly, because these effects did not survive FDR correction (all *q* ≥ 0.050), they are reported as exploratory observations that require independent replication before any confirmatory conclusions can be drawn. For other clinical scales, nominally significant group differences were observed for loneliness (*β* = 0.380, *p* = 0.021, FDR *q* = 0.083) and rumination (*β* = 0.379, *p* = 0.029, FDR *q* = 0.117) regarding posterior Degree, but these effects also did not survive FDR correction.

Collectively, these findings do not provide statistically significant evidence for group-specific brain–resilience associations after correction for multiple comparisons. The consistency of the directionality across multiple topological metrics at the nominal level, however, suggests that this brain–resilience relationship may merit further investigation in larger, independent cohorts. In contrast, the deviation index analysis presented in Section 4.4 yielded associations that remained significant after FDR correction (all *q* < 0.05), which we interpret as the more robust finding of this study.

### 4.4 Exploratory analysis: association between psychological resilience–brain network decoupling and depressive symptoms

To further elucidate the potential clinical implications of these findings, we conducted an exploratory analysis. A healthy reference model was established in the control group to characterize the joint association pattern between regional network features and resilience, and a deviation index was then calculated in the depressed group as the predicted resilience score minus the observed score. Higher values therefore indicated greater deviation from the healthy reference association pattern.

**Figure 4:**
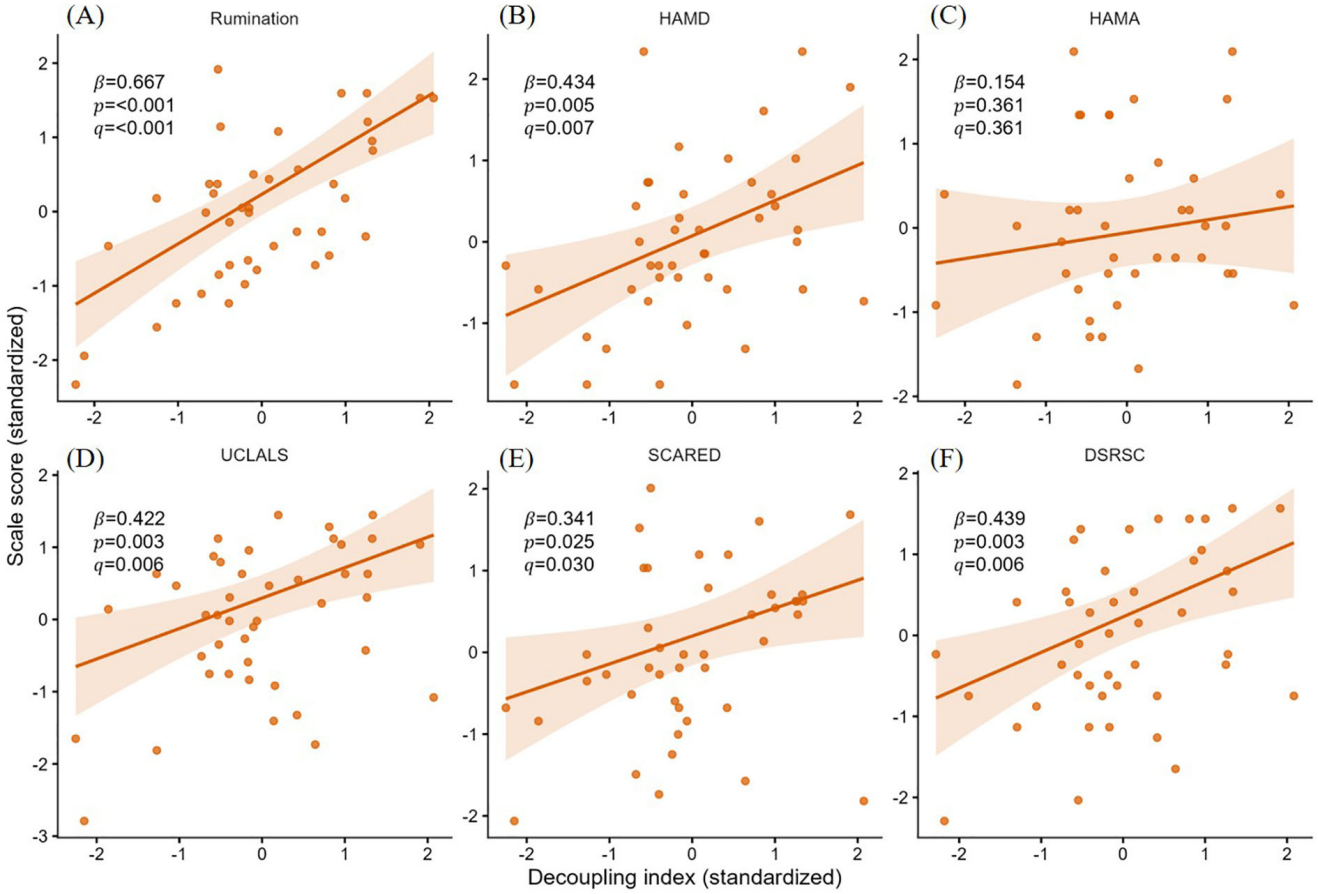
Group scatter plots combined with trend lines show the relationship between the decoupling index (standardized, x-axis) and the scores of six psychological scales (standardized, y-axis): **(A)** Mindfulness Scale, **(B)** HAMD, **(C)** HAMA, **(D)** UCLALS, **(E)** SCARED, **(F)** DSRSC. Each panel shows the data points, the regression line, and the shaded confidence interval of the data points, and reports the β, p, and q values of the measured association.

Within the depression group, this deviation index showed statistically significant positive correlations with multiple symptom dimensions after FDR correction: rumination (*β* = 0.667, FDR *q* < 0.001), self-rated depressive symptoms (DSRSC: *β* = 0.439, FDR *q* = 0.006), clinician-rated depression severity (HAMD: *β* = 0.434, FDR *q* = 0.007), loneliness (R-UCLA-LS: *β* = 0.422, FDR *q* = 0.006), and self-rated anxiety (SCAS: *β* = 0.341, FDR *q* = 0.030), but not clinician-rated anxiety (HAMA: *β* = 0.234, *p* = 0.140, FDR *q* = 0.210) (**Figure 4**). These findings indicate that greater deviation from the healthy brain–resilience reference pattern is associated with a heavier multidimensional symptom burden among depressed adolescents. Notably, the deviation index—quantifying the extent to which each patient deviated from the healthy brain–resilience reference pattern—showed robust and statistically significant correlations with multiple symptom dimensions (all FDR *q* < 0.05). These findings suggest that the brain network features extracted by MVA-GCN, although not showing significant group-wise interactions, capture clinically meaningful information relevant to the heterogeneity of symptom burden in adolescent depression.

## 5 Discussion

This study proposes an innovative Multi-view Adaptive Graph Convolutional Network (MVA-GCN) for adolescent depression screening, which addresses limitations of traditional EEG analysis in feature extraction and information integration. By comprehensively utilizing PLV, PCC, and PLI as parallel graph views combined through adaptive fusion, the model captures complex brain network interactions more comprehensively than single-view approaches. The architecture comprises four core components: multi-view adjacency matrix construction, graph-convolutional layers, adaptive fusion, and a classification module.

In the present sample, questionnaire-based and conventional machine-learning models already showed good discriminative performance, whereas MVA-GCN showed improved performance over the standard GCN model in the present dataset. The near-perfect classification accuracy achieved in the present dataset requires cautious interpretation. First, although sliding-window segmentation generated 134 EEG epochs per participant, this increased the number of model inputs but not the number of independent participants (74 in total). Second, the data came from a single center with consistent equipment and procedures; while this reduces noise, it may also allow the model to learn dataset-specific signal patterns rather than generalizable features. Notably, such high performance is not entirely unprecedented in EEG-based depression detection studies. For instance, Ay et al. (48) reported 99.12% accuracy using a CNN-LSTM model, and Zhang et al. (49) achieved 99.61% accuracy using only four sleep EEG channels, yet both studies acknowledged that their systems were not deployed in real clinical settings. A recent systematic review of 42 studies further reported that classification accuracy ranged from approximately 76% to 100%, and critically noted that near-perfect performance was frequently observed in studies with small sample sizes and subject-dependent or exclusively internal validation strategies, raising concerns regarding overfitting and limited generalizability (50). Additionally, previous resting-state EEG studies using conventional machine learning or single-view deep learning approaches have typically reported substantially lower accuracies compared to the near-perfect performance observed in our study, whereas our MVA-GCN achieved 99.84% in the present dataset. However, direct comparisons across studies are complicated by differences in sample characteristics, EEG protocols, and evaluation frameworks, and the present results should therefore be interpreted with caution. In this context, while the performance of MVA-GCN is encouraging, it should be regarded as a preliminary proof-of-concept finding rather than evidence of established clinical utility. Future studies with independent external validation across centers, devices, and larger samples are critically needed to evaluate the stability and generalizability of the model.

Beyond merely reporting superior model performance, this study advances the field by bridging computational outputs with clinical phenotypes. We identified ten high-contributing nodes, which were spatially aggregated into two candidate clusters: the posterior and fronto-central regions. We then examined whether the topological metrics of these regions showed group-dependent associations with psychological resilience and other clinical dimensions. After rigorously controlling for age and sex and applying FDR correction for multiple comparisons, none of the Group × Network feature interaction effects reached statistical significance (all q ≥ 0.050). The resilience-related tests yielded the lowest q values among all scales examined (*q* = 0.050), suggesting that this dimension may warrant further investigation in future studies, but these findings do not constitute statistically significant evidence in the present sample.

Importantly, the absence of significant interaction effects does not imply that the MVA-GCN-derived network features lack clinical relevance. Rather than focusing on case–control differences in brain–behavior associations, we adopted a complementary approach: we constructed a healthy reference model based on the control group and computed a deviation index for each patient, quantifying the extent to which each individual’s brain–resilience association pattern deviated from the healthy normative pattern. As detailed in Section 4.4, this deviation index showed robust and statistically significant correlations with multiple symptom dimensions after FDR correction (all *q* < 0.05). These findings suggest that the brain network patterns captured by the MVA-GCN model may reflect clinically meaningful variations in symptom burden among depressed adolescents—specifically, greater deviation from the healthy brain–resilience reference pattern is associated with more severe rumination, depression, loneliness, and anxiety.

Prior studies have similarly implicated the functional architecture of fronto-parietal, prefrontal, and occipital networks in diverse psychological symptoms and emotional processing (51–55), including childhood trauma, depression, and facial emotion recognition. The variability in specific scale associations across studies likely stems from the functional heterogeneity within these regions (56–58). In our study, the nominal-level correlational patterns between nodal topological indices and resilience, although not surviving multiple comparison correction, provide exploratory descriptive information that may inform hypothesis generation for future investigations. However, these patterns do not by themselves establish a direct neurobiological mechanism.

Building on these insights, we constructed a healthy reference model based on the control group to define a deviation index for each patient, quantifying the extent to which each individual’s brain–resilience association pattern deviated from the healthy normative pattern. Within the MDD group, this deviation index showed statistically significant positive correlations with multiple symptom dimensions after FDR correction: rumination (*β* = 0.667, FDR *q* < 0.001), self-rated depression (DSRSC: *β* = 0.439, FDR *q* = 0.006), clinician-rated depression severity (HAMD: *β* = 0.434, FDR *q* = 0.007), loneliness (R-UCLA-LS: *β* = 0.422, FDR *q* = 0.006), and self-rated anxiety (SCAS: *β* = 0.341, FDR *q* = 0.030), but not clinician-rated anxiety (HAMA: *β* = 0.234, p = 0.140, FDR *q* = 0.210). These findings indicate that greater deviation from the healthy brain–resilience reference pattern is associated with a heavier multidimensional symptom burden among depressed adolescents.

It is crucial to note, however, that this deviation analysis is exploratory in nature and based on a cross-sectional design; thus, it establishes correlational associations rather than causal relationships. Nevertheless, these results provide a clear conceptual bridge: the features extracted by the MVA-GCN model are not merely computationally advantageous for classification but may correspond to clinically interpretable dimensions, such as individual differences in resilience-relevant functioning and symptom severity.

## 6 Limitation

Several limitations warrant consideration.

First, the modest sample size and the absence of an independent external validation cohort indicate that both the classification performance and the clinical correlation findings should be interpreted as preliminary.

Second, the clinical interpretability analysis focused on high-contribution nodes and employed strategies such as regional aggregation and selective scale analysis. Although these approaches enhanced clarity, they also render the current findings exploratory in nature and in need of replication.

Third, the cross-sectional design precludes inferences regarding the temporal sequence or causality between brain network alterations and symptom manifestation.

Fourth, although the primary aim of this study was to explore the discriminative potential of a multi-view brain network framework rather than to isolate medication effects, we acknowledge that concurrent pharmacotherapy may have influenced the EEG metrics. Psychotropic drugs are known to exert substantial effects on resting-state EEG power spectra, functional connectivity, and graph-theoretical network properties (59, 60). The heterogeneity in medication status across patients thus represents a potential confound in the interpretability of our findings, and the observed nominal-level brain–resilience association patterns and their clinical correlates should therefore be interpreted with caution. Future studies with larger samples are warranted to systematically collect standardized medication information and incorporate these variables as covariates in the analytical models. Alternatively, recruiting first-episode, medication-naïve patient cohorts would help clarify whether the identified EEG network features represent intrinsic depression-related alterations independent of medication effects.

Fifth, although we provided descriptive comparisons of classification performance across multiple models (**Tables 2**, **3**) using a unified experimental framework, we did not perform formal statistical tests to compare classifiers (e.g., paired tests across cross-validation folds or the Nemenyi *post-hoc* test following Friedman’s test). While the performance gaps between MVA-GCN and the competing models substantially exceeded the cross-validation variability observed within each model, formal statistical comparisons would provide stronger evidence for the superiority of one model over another. However, we note that the validity of such tests in cross-validation settings requires careful consideration, as the performance estimates from different folds are not fully independent (61, 62).

Future studies with larger, independent cohorts should conduct more formal statistical benchmarking to confirm the generalizability of these findings. In addition to these limitations, future work should validate the stability and generalizability of the multi-view brain network model in larger, longitudinal, and independent samples, to determine whether it can provide reliable support for clinical screening, stratified assessment, and treatment response monitoring.

## 7 Conclusion

In summary, the MVA-GCN model based on multi-view resting-state EEG functional connectivity showed promising proof-of-concept discriminative capability for adolescent depression in the present sample. The observed resilience-related deviation from the healthy reference association pattern among depressed adolescents showed significant correlations with aggravated clinical emotional symptoms after FDR correction. These exploratory findings suggest that complementary usage of objective EEG brain network detection and traditional self-report scales has potential value for auxiliary screening, but the proposed framework should not yet be regarded as a clinically validated diagnostic tool in Asian adolescent clinical settings.

## Data Availability

The raw data supporting the conclusions of this article will be made available by the authors, without undue reservation.
